# Signatures of the Giant Pairing Vibration in the ^14^C and ^15^C atomic nuclei

**DOI:** 10.1038/ncomms7743

**Published:** 2015-03-27

**Authors:** F. Cappuzzello, D. Carbone, M. Cavallaro, M. Bondì, C. Agodi, F. Azaiez, A. Bonaccorso, A. Cunsolo, L. Fortunato, A. Foti, S. Franchoo, E. Khan, R. Linares, J. Lubian, J. A. Scarpaci, A. Vitturi

**Affiliations:** 1 Dipartimento di Fisica e Astronomia, Università di Catania, via S. Sofia 64, I-95125 Catania, Italy; 2 Istituto Nazionale di Fisica Nucleare, Laboratori Nazionali del Sud, I-95125 Catania, Italy; 3 Institut de Physique Nucléaire, Université Paris-Sud-11-CNRS/IN2P3, 91406 Orsay, France; 4 Istituto Nazionale di Fisica Nucleare, Sezione di Pisa, I-56127 Pisa, Italy; 5 Dipartimento di Fisica e Astronomia, Università di Padova, I-35131 Padova, Italy; 6 Istituto Nazionale di Fisica Nucleare, Sezione di Padova, I-35131 Padova, Italy; 7 Istituto Nazionale di Fisica Nucleare, Sezione di Catania, Via S. Sofia 64, I-95125 Catania, Italy; 8 Instituto de Física da Universidade Federal Fluminense, 24210-346 Rio de Janeiro, Niterói, RJ, Brazil; 9 Centre de Sciences Nucléaires et de Sciences de la Matières - CSNSM, Université Paris-Sud-11-CNRS/IN2P3, 91405 Orsay, France

## Abstract

Giant resonances are collective excitation modes for many-body systems of fermions governed by a mean field, such as the atomic nuclei. The microscopic origin of such modes is the coherence among elementary particle-hole excitations, where a particle is promoted from an occupied state below the Fermi level (hole) to an empty one above the Fermi level (particle). The same coherence is also predicted for the particle–particle and the hole–hole excitations, because of the basic quantum symmetry between particles and holes. In nuclear physics, the giant modes have been widely reported for the particle–hole sector but, despite several attempts, there is no precedent in the particle–particle and hole–hole ones, thus making questionable the aforementioned symmetry assumption. Here we provide experimental indications of the Giant Pairing Vibration, which is the leading particle–particle giant mode. An immediate implication of it is the validation of the particle–hole symmetry.

## Abstract (web summary)

The Giant Pairing Vibration is a collective mode in an atomic nucleus caused by coherence between particle-particle excitations, which has so far eluded detection. Cappuzzello *et al*. present signatures for its existence via heavy-ion-induced two-neutron transfer reactions in carbon nuclei.

## Graphical abstract

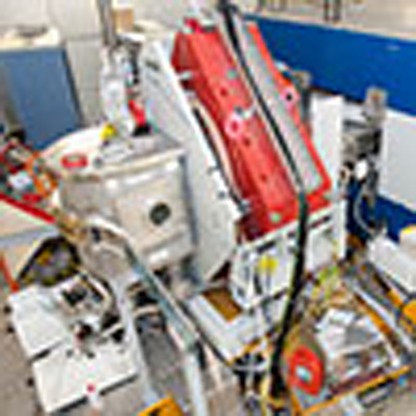

In 1977, Broglia and Bes^1^ predicted the existence of a giant collective mode in the atomic nuclei, named the Giant Pairing Vibration (GPV). The microscopic origin of such a mode is the coherence among elementary particle–particle excitations generated by the addition or removal to/from an atomic nucleus of two nucleons in a relative *S*-wave (motion component characterized by an orbital angular momentum *L*=0). This quantum mechanism is analogous to that feeding the giant resonances (GR) observed in the inelastic nuclear excitation. The link between GPV and GR is the symmetry between single-particle states above (particle) and below (hole) the Fermi level, assumed for quantum systems of fermions such as the nuclei. In both cases the only required condition is a system whose minimum energy structure is governed by a mean field. This is the case of atomic nuclei, in which the mean field gives the most reliable representation of its many-body nature. GR are collective states, manifestation of a coherence mechanism of the particle–hole (*p*–*h*) excitations connecting major shells of the harmonic oscillator-like nuclear mean field. The properties of the observed GR are determined by the residual *p*–*h* interaction, that is, the small deviation of the actual potential felt by the particles in the excited state from the mean field in the ground state. In a symmetric manner the GPV represents the particle–particle (*p*–*p*) or the hole–hole (*h*–*h*) counterpart of the GR. The main component of the *p*–*p* or *h*–*h* residual interaction is the pairing field, which is known to be attractive and in a relative spin-less *S*-wave. The introduction of such a field explains why at the minimum energy the nuclei with an even numbers of neutrons and protons are more tightly bound than the neighbouring even-odd or odd-odd ones.

In recent decades, the theory has refined the initial approach with the introduction of microscopically derived form factors^2^,^3^,^4^. However, this has produced only minor changes in terms of the estimation of the GPV excitation energy, multipolarity and strength. Historically, the theoretical predictions were mainly limited to the Sn and Pb even–even isotopes, since the effects coming from the pairing condensate (Sn) or shell closure (Pb) can be disentangled more clearly^5^,^6^. Nevertheless, recent continuum quasi-particle random phase approximation (cQRPA) calculations concerning oxygen isotopes^7^,^8^,^9^ have shown that the GPV are also expected in light nuclei. Quantitatively, these modes are predicted to lie at an excitation energy of ~20 MeV with respect to the unperturbed system, to have a width of ~1–2 MeV, an *L*=0 character and a high degree of collectivity.

Despite the existence of the GPV mode has been theoretically suggested and several evidences of *p*-*h* giant modes have been observed, no resonances with the properties defining the GPV has been identified in any nucleus so far^10^,^11^,^12^. This is probably due to the particular reaction mechanism required for populating the GPV. The addition or removal of two nucleons can be obtained in the laboratory by two-nucleon transfer reactions. These processes are typically characterized by a sizeable exchange of linear momentum at the surface of the colliding nuclei. Consequently, the angular momentum transferred in the reaction tends to be large and the cross-section for the *L*=0 modes is expected to be suppressed. This feature is more evident for reactions induced by heavy-ion projectiles and characterized by large-negative *Q*-values (difference between the mass of the initial and final colliding systems)^13^.

Herein we report on an experimental approach to investigate the GPV mode, based on heavy-ion-induced two-neutron transfer reactions on light nuclei. The ^12^C(^18^O,^16^O)^14^C and ^13^C(^18^O,^16^O)^15^C reactions at 84 MeV incident laboratory energy are studied. A set of signatures that can indicate the population of the GPV is found, which represents a significant progress in this research field. In the ^14^C nucleus the GPV is identified at an excitation energy of 19.9±0.1 MeV with respect to the target ground state and in the ^15^C one at 20.4±0.1 MeV. The measured widths are 1.2±0.3 MeV and 1.9±0.3 MeV (full-width at half-maximum) for ^14^C and ^15^C, respectively. The *L*=0 nature of the transition to these resonances is suggested by model-independent analysis of the measured cross-section angular distributions. The experimental 0^+^ response is well described by microscopic calculations of the nuclear response based on cQRPA. State-of-the-art calculations of the reaction cross-section for the transition to bound and continuum states of the residual support the *L*=0 character of the transition. The extracted transfer probabilities for the *L*=0 transitions are also consistent with the GPV population.

## Results

### Experimental details

The experiment was performed at the INFN-LNS laboratory in Catania. A beam of ^18^O^6+^ ions, extracted by the Tandem Van de Graaff accelerator, at 84 MeV incident energy impinged, in separate runs, on a ^12^C and a ^13^C target. The ^16^O ejectiles produced in the collisions were momentum analysed by the MAGNEX spectrometer^14^,^15^, working in full acceptance mode (solid angle *Ω*~50  msr and momentum bite Δ*p*/*p*~24%). The ejectiles were identified event by event in atomic number (*Z*), atomic mass (*A*) and charge (*q*)^16^ (see Methods and Supplementary Fig. 1). The trajectories of the ^16^O^8+^ ejectiles were then reconstructed and the momentum vector was deduced at the target position^17^ (more details are given in the Methods section).

### Spectroscopic features of the populated states

The reaction *Q* values or, equivalently, the excitation energies *E*_x_=*Q*_0_−*Q* (where *Q*_0_ is the ground-to-ground state *Q* value) were obtained by the missing mass determination based on relativistic kinematic transformations. The excitation energy compared with the target ground state *E*^t^_x_=*E*_x_+*M*_r_−*M*_t_ was also computed, where *M*_t_ and *M*_r_ represent the mass of target and residue, respectively. The *E*^t^_x_ is a more suitable parameter when comparing the data with theoretically derived *p*–*p* excitations built on the target mean field. An overall energy resolution of ~160 keV full-width at half-maximum (FWHM) is obtained from Gaussian fit of the observed transitions to bound states of the residual nuclei. The laboratory angle resolution is better than 0.5° and the accuracy is estimated within 20 keV in energy and 0.05° in laboratory angle.

**Figure 1:**
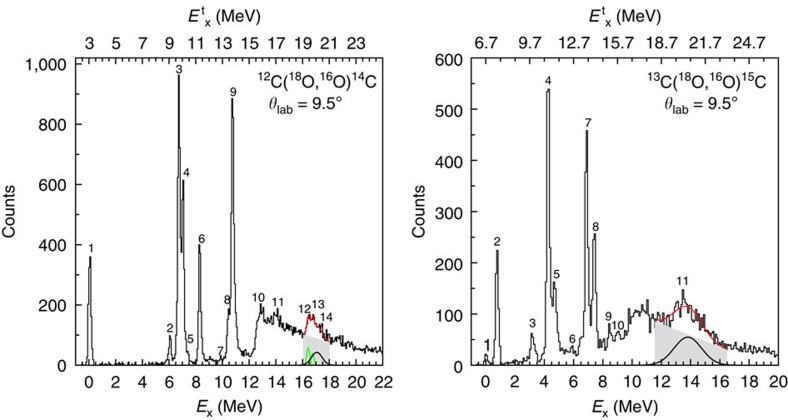
Energy spectra for the ^12^C(^18^O,^16^O) and the ^13^C(^18^O,^16^O) reactions at 84 MeV at forward angles. The linear background models, the fitted bumps and their sum are shown as the grey area, the black Gaussian and the red continuous line, respectively. In the ^14^C case, the known resonances at 16.43 and 16.72 MeV are also indicated (green Gaussians).

Examples of the reconstructed energy spectra for the two experiments are shown in Fig. 1. Several narrow peaks corresponding to well-known low-lying bound and resonant states are observed in both cases, as listed in Table 1. A sudden increase of the yield is also found just above the two-neutron separation energy (*S*_2n_=13.123 MeV for ^14^C and *S*_2n_=9.394 MeV for ^15^C). This observation is discussed in the Methods section, in which two extreme models describing the effect in the energy spectra of two-neutron removal from the projectile (projectile break-up) are presented. A large bump, superimposed to the continuum background, is observed in both spectra. A best fit procedure with Gaussian shapes and a locally adjusted linear background model (see Fig. 1) gives an energy of *E*_x_=16.9±0.1 MeV (FWHM 1.2±0.3 MeV) in ^14^C and *E*_x_=13.7±0.1 MeV (FWHM 1.9±0.3 MeV) in ^15^C. Different model functions do not change the obtained results within the quoted uncertainties. In the ^14^C case two known narrow resonances at 16.43 and 16.72 MeV are weakly populated in the lower-energy region of the bump and accounted for in the fit. The centroids and widths are confirmed by a supplementary experiment performed at 270 MeV bombarding energy studying the same reactions by the same experimental set-up. These bumps do not correspond to any structures previously reported and are not explained by the projectile excitation or break-up contribution to the energy spectra^18^ (see Methods section and Supplementary Fig. 2).

### Absolute cross-section angular distributions

**Figure 2:**
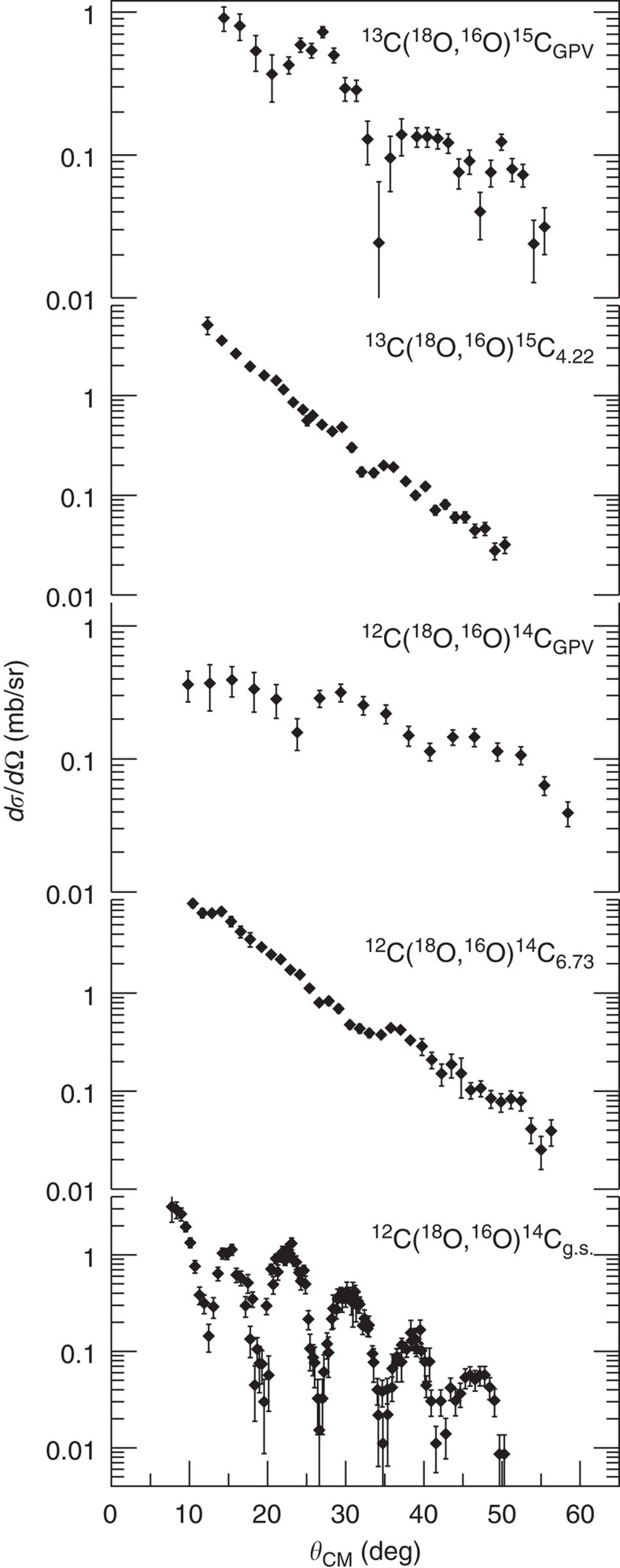
Angular distributions for the ^12^C(^18^O,^16^O)^14^C and ^13^C(^18^O,^16^O)^15^C reactions at 84 MeV. The error bars correspond to the combination of uncertainties coming from the solid angle determination, the statistical error and the background subtraction (see text).

The absolute cross-section angular distributions were extracted, according to the procedure described in ref. 19. This allowed an accurate treatment of key aspects such as the solid angle estimation and the transport efficiency of the spectrometer^20^. The background underneath each peak was modelled by a linear function, and the possible overlap between close peaks was treated by a least-squared analysis assuming Gaussian shapes. The background due to ^12^C impurities in the ^13^C target was also taken into account. The experimental angular distributions for the transitions to the ^14^C 0^+^ ground and 3^−^ excited states and for the ^15^C 5/2^−^ one are compared with those of the broad bumps at 16.9±0.1 MeV in ^14^C and 13.7±0.1 MeV in ^15^C in Fig. 2. The contribution to the angular distribution of the bumps due to the continuous background in the spectra was estimated at each angle by a least-squared approach. The analysis of such a background is reported in the Supplementary Methods, where different models for the background subtraction are compared (see Supplementary Figs 3–5). The obtained results for the centroid and width of the resonances and also the shape of the angular distributions do not change within the quoted uncertainties. A cross-section scale error of approximately±10%, mainly generated by uncertainties on the target thickness, is common to all the points of the angular distributions and it is not included in the error bars shown in Fig. 2. These correspond to other sources of uncertainty such as the solid angle determination (~2%), the statistical error (~6%) and the background subtraction. The last-named is the leading term for the broad bumps overlapping in the continuum (~30%).

A clear indication of an oscillating pattern is present only in the ^14^C 0^+^ ground state and in the two broad bumps. An analysis of the angular distribution shape for the ^14^C bump is reported in the Supplementary Methods and Supplementary Fig. 6. This striking behaviour reveals the dominance of a resonant state in that energy region, characterized by a well-defined angular momentum. The properties of such resonances will be analysed and compared with those expected for the GPV.

### Energy of the resonances

A first similarity between the two resonances is suggested by the *E*^t^_x_ representation (see Fig. 1), in which they are located at *E*^t^_x_=19.9±0.1 MeV in ^14^C and *E*_x_^t^=20.4±0.1 MeV in ^15^C. It is worth noticing that in carbon isotopes the 2*s*_1/2_ single-particle orbital (representing a configuration whose wave function has *n*=2 radial nodes, *l*=0 orbital angular momentum and *j*=1/2 total angular momentum) is found at similar single-particle energy (within 1 MeV or less) as the 1*d*_5/2_ one (*n*=1, *l*=2, *j*=5/2), while the 1*d*_3/2_ (*n*=1, *l*=2, *j*=3/2) is at about 5 MeV from the former. An estimate of the two-neutron 0^+^ states built on such orbitals would predict a doublet with [1*d*_5/2_⊗1*d*_5/2_]_0+_ (representing a configuration with two neutrons in the 1*d*_5/2_ orbital coupled to 0^+^ overall angular momentum) and [2*s*_1/2_⊗2*s*_1/2_]_0+_ configurations at low excitation energy and an isolated state with a [1*d*_3/2_⊗1*d*_3/2_]_0+_ configuration ~10 MeV over the doublet. The introduction of the residual neutron–neutron interaction generates a significant mixing between the [1*d*_5/2_⊗1*d*_5/2_]_0+_ and [2*s*_1/2_⊗2*s*_1/2_]_0+_ configurations^21^ with a minor influence on the high-lying [1*d*_3/2_⊗1*d*_3/2_]_0+_ state. This has been partially demonstrated in the case of ^14^C in ref. 22 where the two low-lying 0^+^ states are found at 6.577 and 9.746 MeV. In this work the population of the afore-mentioned states is strongly suppressed compared with the 16.9±0.1 MeV one (see Fig. 1). The *L*=0 character of this resonance, discussed in the next section, suggests a relevant degree of collectivity in the region where the [1*d*_3/2_⊗1*d*_3/2_]_0+_ is expected to dominate. An analogous situation is expected for ^15^C, where the corresponding of the ^14^C 0^+^ low-lying states are at 3.103 and 5.866 MeV. Our results have highlighted a much reduced cross-section for such states compared with the much stronger populated resonance at 13.7±0.1 MeV, that is, about 10 MeV above the state at 3.103 MeV.

The observed resonances are compatible with the above-mentioned prediction of *p*–*p* cQRPA calculations in oxygen isotopes^7^. To ascertain this, the same kind of calculations was performed for the ^12^C response to the monopole *p*–*p* operator to predict the 0^+^ two neutrons addition strength. This approach represents the state-of-the-art for the extraction of the *p*–*p* response function. However, the assumed spherical symmetry and the mean-field approximation may not be optimal for light nuclei such as ^12^C. A broad distribution connected to the superposition of the 2*p*_3/2_, 2*p*_1/2_, 1*d*_3/2_, 1*f*_7/2_ and orbitals above is identified in the response function at *E*_x_^theor^~17 MeV with respect to the ^14^C_g.s._, a value consistent with the experimental centroid of the bump (*E*_x_=16.9±0.1 MeV). A certain degree of collectivity is observed in such bump when the residual interaction is taken into account. Such a prediction is a first argument in favour of the identification of the experimental resonance as the GPV.

It is also worth that the measured widths are consistent with the discussion about the GPV of refs 12, 23 and are of the same order of the measured GR ones in such light nuclei^24^. The different width in the two systems indicates a different half-life, which is shorter in the case of ^15^C. This is mainly due the fact that the ^15^C resonance is about 4.3 MeV above *S*_2n_ and 12.5 MeV above the one-neutron emission threshold (*S*_n_), whereas ^14^C resonance is ~3.7 MeV above *S*_2n_ and 8.7 MeV above *S*_n_. The larger energy available in ^15^C results in a substantially faster emission of both one and two neutrons.

### Multipolarity of the resonances

Two approaches are used to investigate the multipolarity of the observed resonances: (i) the analysis of the oscillating behaviour of the experimental angular distributions, (ii) the comparison of the measured cross-sections with state-of-the-art theoretical calculations.

A well-known phenomenon in heavy-ion-induced transfer reactions above the Coulomb barrier is the presence of oscillations in the angular distributions only for the *L*=0 transitions^13^. Semi-classical and fully quantum-mechanical models predict this property^13^,^25^. The distinctive feature of the *L*=0 angular distributions provides a model-independent identification of *L*=0 among the other multipolarities. This phenomenon was experimentally observed for the same ^12^C(^18^O,^16^O)^14^C reaction at 84 MeV in ref. 25 for transitions below *S*_2n_. In the present case, the oscillating pattern of the broad resonances angular distributions supports their dominant *L*=0 nature (see Fig. 2). The different period of the observed oscillations with respect to the ^14^C 0^+^ ground state is a direct consequence of the higher excitation energy of the investigated resonances, which determines a reduction of the wavenumber in the outgoing channel.

**Figure 3:**
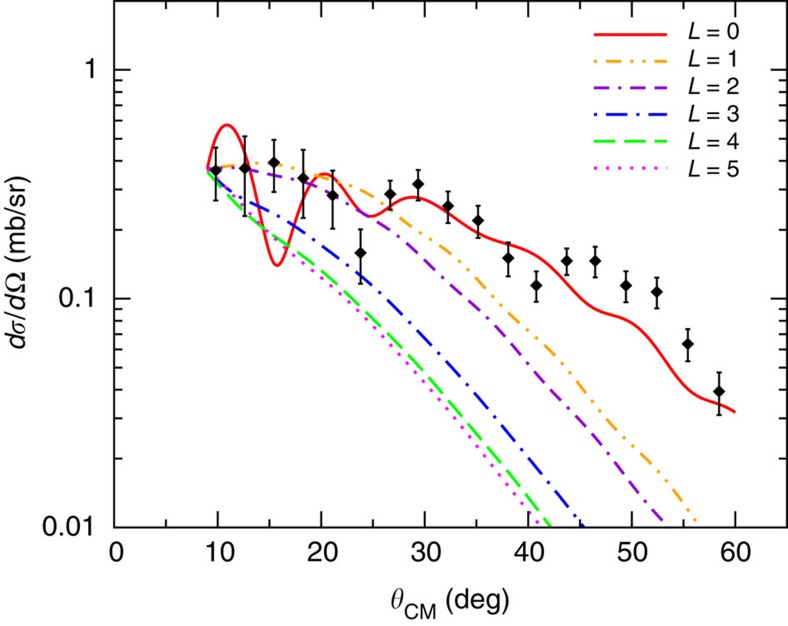
Comparison between the CRC calculations and the measured cross section for the ^14^C resonance at 16.9 MeV. The calculations are performed at 12 MeV excitation energy for *L*=0 to 5 and are normalized to coincide at *θ*_CM_=9°. The error bars correspond to the combination of uncertainties coming from the solid angle determination, the statistical error and the background subtraction (see text).

Coupled reaction channel (CRC) calculations have also been performed to infer the multipolarity of the resonant mode in ^14^C. The São Paulo parameter free double folding potential was used for the real part in the optical model potential^26^. The imaginary part was taken from ref. 27, which is known to give a good description of absorption for tightly bound systems. The calculations were performed assuming an extreme cluster model approximation for the two neutrons. The wave functions used in the form factor calculations were generated by a Wood–Saxon-shaped potential, whose depth was chosen to reproduce the experimental binding energies for two neutrons. The same ingredients were employed in ref. 25. The present calculations were separately performed for various *L* components from *L*=0 to 5 and excitation energies below *S*_2n_. Above this threshold, CRC calculations are unstable. The results are reported in Fig. 3 assuming a transition to a ^14^C resonance at 12 MeV. This energy represents the closest value to the actual ^14^C GPV, which guarantees a stable convergence of the calculations. In the absence of reliable spectroscopic amplitudes for this transition, the calculations have been normalized to coincide at *θ*_CM_=9°, thus only the shape can be discussed. The calculated angular distributions do not present oscillating behaviour, except the *L*=0 one. Moreover, the *L*=0 gives the best representation of the experimental slope. Such a result gives additional indication in favour of the *L*=0 nature of the transition to the ^14^C resonance.

When considering a resonance above the two-neutron emission threshold, a proper approach for the cross-section calculation would be the one proposed in ref. 28, in which transitions to unbound states are described in a discretized scheme. However, state-of-the-art calculations in this framework are nowadays limited to three-body assumption, whereas the discussed problem would require a full four-body approach. As a consequence, finer details of the calculations could not be sufficiently accurate and only the global features can be considered. This kind of approach was applied for describing the cross-section of the ^14^C resonance. Details concerning the calculations and the results are reported in the Supplementary Methods and Supplementary Fig. 7. The absolute value for *L*=0 transition is found to be consistent with the experimental one, without the need of any scaling factor.

### Strength of the resonances

To extract the strength of the resonances, the measured cross-sections were factorized into the scattering cross-section (*dσ/d*Ω)_sc_, the transfer probability *P*_tr_(*θ*) and quantal corrections *F*(*Q*,*L*)


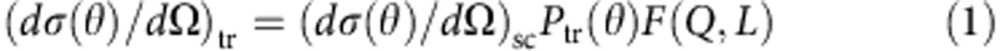


according to ref. 23. Such an approximation is known to be appropriate when the relative motion can be described in a semi-classical approach, as in the present case (short wavelength *λ*~0.1 fm compared with the radii of the involved nuclei and minor role of the Coulomb field, as a consequence of a Sommerfeld parameter *η*=3.5). The quantal correction factor *F*(*Q*, *L*) corresponds to two different matching aspects: one related to the scattering orbits and the other to the internal matching of the bound-state wavefunctions. Considering only the *L*=0 transitions, such a factor is:


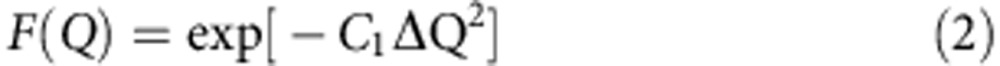


where *C*_1_ was evaluated according to ref. 23 and Δ*Q* according to ref. 29. The scattering cross-section (*dσ/d*Ω)_sc_ was calculated in a CRC framework using the São Paulo potential. As discussed in ref. 1, the GPV strength is predicted to be similar to the strength of the *L*=0 transition to the ground state in Pb and Sn even–even isotopes. To verify this property, we compared the ^14^C GPV *P*_tr_ with that of the ground-state transition, obtaining a ratio 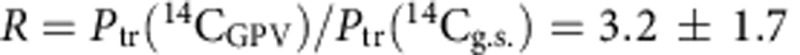. In the ^15^C case, as the *L*=0 mode to the lowest harmonic oscillator shell (1*p*) is not allowed, we extracted the ratio 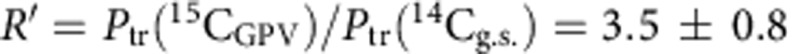. The uncertainties are due to both theoretical and experimental sources. In both instances, the GPV transfer probability is consistent with the theoretical predictions. The larger cross-section for GPVs is probably due to their larger phase space of single-particle orbitals compared with the ^14^C_g.s._ one. Indeed, taking into account the degeneracy in magnetic substates, the GPV 0^+^ space includes at least nine configurations, built within the 2*p*_3/2_, 2*p*_1/2_, 1*d*_3/2_, 1*f*_7/2_ orbitals. These contribute independently to the outgoing flux in the GPV channel. Otherwise, only three 0^+^ configurations are possible for the ^14^C_g.s._, within the 1*p*_3/2_, 1*p*_1/2_ space, consistently with the measured ratios for the transfer probabilities.

## Discussion

Our data show the clearest signal compatible with the long-searched GPV so far. The resonances at *E*_x_=16.9±0.1 MeV in ^14^C and at *E*_x_=13.7±0.1 MeV in ^15^C show properties consistent with those defining the GPV mode. If the signals observed are indeed due to the excitation of the GPV, they confirm the foundation of the particle–hole quantum symmetry in a system of interacting fermions and can provide benchmark information on the role of the pairing field in nuclear structure. A microscopic description of such a field is essential to gain a much deeper understanding of a wide class of nuclear matter phenomena observed in the exploration of many-body systems, from nuclear chart to neutron stars. Further work is needed to confirm the results and provide clear evidence for the observation of the GPV. It is worth exploring other systems, where structural and reaction mechanism constraints are as favourable for the GPV excitation as the present case. Moreover, the study of the decaying features of these resonances could reveal further information on the nature of the neutron–neutron correlation in the giant mode.

## Methods

### Experimental set-up

The ^14^C and ^15^C nuclei were populated through the (^18^O,^16^O) two-neutron transfer reaction. A 49±3 μg cm^−2^ self-supporting ^12^C and a 50±3 μg cm^−2^ self-supporting 99% enriched ^13^C targets were used. The ^18^O^6+^ beam at 84 MeV incident energy was produced and accelerated by the Tandem Van de Graaff facility of INFN-LNS with a current ranging from 10 to 50 enA. The beam spot size at the target, defined by a collimation system, was 1.2 mm horizontal and 2.3 mm vertical. The horizontal angular divergence was ~0.8 mrad, whereas the vertical one was ~3 mrad.

A 8-mm-diameter Faraday cup was located in the scattering chamber 15 cm downstream the target along the incident beam direction. The beam current integrated by the cup gave a measurement of the overall charge at each acquisition run with an accuracy better than 1%.

The ^16^O ejectiles were momentum analysed by the MAGNEX spectrometer, covering a solid angle Ω~50 msr and momentum acceptance Δ*p*/*p*~24%. The explored angular range was between 3° and 24° in the laboratory reference frame. The accuracy in the angle determination was better than 0.05°.

### MAGNEX and the focal plane detector setting

The MAGNEX large acceptance magnetic spectrometer^14^,^15^ is a high-performance device, installed at the INFN-LNS, offering a high-energy, angular and mass resolution in a large-accepted phase space. MAGNEX is a QD spectrometer consisting of a vertically focusing quadrupole magnet (Q) and a 55° bending magnet (D) providing the dispersion and the horizontal focusing strength.

The magnetic field of the MAGNEX dipole was set to horizontally focus the ^16^O^8+^ ejectiles corresponding to the ^14^C_g.s._ (or ^15^C_g.s._) 8% larger rigidity from the optical axis. The quadrupole field was set to ensure that the vertical focus is achieved at the interception between the optical axis and the focal plane.

The MAGNEX focal plane detector (FPD)^30^ consists of a proportional drift chamber divided in five sections, four of which are position-sensitive, and a wall of stopping silicon detectors at the back. It measures the horizontal and vertical positions of each incident ion at four successive planes along the ion trajectory. Also, it measures the energy loss in the gas and the residual energy released in the silicon detectors. The FPD was filled with isobutane gas (99.95% pure) at 7 mbar pressure. A Mylar entrance window of 920 × 220 mm^2^ area and 1.5 μm thickness contained the gas. The proportional counter section includes five sets of amplifying wires. A set of 224 induction pads orientated along the spectrometer optical axis is located 5 mm above each proportional wire. The centre of gravity of the charge distribution at each counter section gives the horizontal position *X*_i_ (ref. 31). Sixty silicon pad detectors, arranged in 20 columns and 3 rows, are located at the back of the gas detector.

### Particle identification

The ejectiles are identified by the measurement of energy loss (Δ*E*), residual energy (*E*_resid_), horizontal position (*X*_foc_) and angle (*θ*_foc_) at the focus, according to the technique described in ref. 16, where the capability of the instrument to identify the oxygen ejectiles was demonstrated. The first step consists in the identification of the atomic number (*Z*) of the ejectiles performed by a standard Δ*E*-*E* technique. A typical Δ*E*-*E* plot is shown in the upper panel of Supplementary Fig. 1 for a single silicon detector together with a coarse graphical contour that includes the oxygen ejectiles. In particular, the plotted parameters are the residual energy measured by the silicon detectors (*E*_resid_), and the energy-loss in the gas measured by the proportional counter, corrected for the trajectory length (Δ*E*_corr_). The oxygen isotope loci in the Δ*E*–*E* plot were identified through supplementary elastic scattering runs. A plot of the two measured quantities (*X*_foc_ and *E*_resid_) used for the mass (*A*) identification is shown in the lower panel of Supplementary Fig. 1, for the data selected with the graphical condition on the Δ*E*_corr_−*E*_resid_ (upper panel of Supplementary Fig. 1). Three main groups are visible corresponding to three different charge states (8^+^, 7^+^ and 6^+^). Inside each group, the different oxygen isotopes are clearly separated (^18^O, ^17^O and ^16^O). The ^16^O^8+^ ejectiles were selected by the graphical contour shown in the lower panel of Supplementary Fig. 1.

### Trajectory reconstruction

The off-line data reduction basically resembles that of ref. 19. The positions and angles of the identified and selected ions in the dispersive and not dispersive directions, measured at the focal plane, were used as input for a 10th order ray-reconstruction, based on the differential algebraic method implemented in MAGNEX^17^.The procedure requires that the object point, the magnetic elements and the FPD positions are known with sub-millimetric precision in the same three-dimensional space. This was obtained by accurate geometrical measurements and alignments using theodolites and bubble level optical devices.

The ray-reconstruction technique allows an effective compensation of the high-order aberrations connected with the large acceptance of the spectrometer. As a result, the initial phase space parameters at the target point are obtained, which are directly related to the momentum modulus and the scattering angle of the detected ejectiles. The laboratory scattering angle is extracted from the initial horizontal and vertical ones by simple geometrical relations, whereas the ejectiles kinetic energy is deduced from the reconstructed momentum. The corresponding *Q* values, or equivalently the excitation energy *E*_x_=*Q*_0_−*Q* (where *Q*_0_ is the ground-to-ground state *Q*-value), are finally obtained by a missing mass determination using relativistic energy and momentum conservation laws, assuming a binary reaction.

The high resolution (0.2° in horizontal angle, 0.7° in vertical angle, 1/1800 in momentum modulus) and accuracy (better than 0.05° in angles and 1/1200 in momentum modulus) in the reconstruction of both the momentum direction and modulus was demonstrated in ref. 17, using a pepper-pot shaped diaphragm that precisely defined few control trajectories.

The capability of the MAGNEX spectrometer to accurately measure absolute cross-sections was demonstrated in ref. 20, where the transmission efficiency of the ejectiles through the spectrometer was studied.

### Projectile break-up calculations

To interpret the background due to break-up processes above the two-neutron separation energy in the inclusive spectra, two independent semi-classical models were used. One assumes the removal of two independent neutrons from the projectile, the other the towing of a di-neutron system. Calculations based on the transfer to the continuum of the target+*n*+*n* resonances do not reproduce these structures when no *n*-*n* correlations are assumed. The adopted method extends the formalism of the transfer to bound states^32^ to the case of unbound ones^33^,^34^. The calculations were performed for the ^13^C(^18^O,^16^O) reaction, which was interpreted as a two-step mechanism: ^18^O+^13^C→^17^O+^14^C_g.s._→^16^O+^14^C_g.s._+*n* starting from the one-neutron separation energy (*S*_*n*_=1.218 MeV) and ^18^O+^13^C→^17^O+^13^C_g.s._+*n*→^16^O+^13^C_g.s._+*n*+*n* starting from the two-neutron separation energy (*S*_2n_=9.394 MeV). The cross-section was calculated within a semi-classical approximation, according to the model described in ref. 34. One of the key ingredients of such formalism is the optical model *S*-matrix, which describes the neutron-target interaction. For the present calculations a constant optical potential from the parameterization given in ref. 35 for *n*+^13^C and *E*=10 MeV was adopted. It has a Woods–Saxon real volume plus a spin-orbit and surface imaginary terms.

The resulting calculation is superimposed to the experimental energy spectrum of ^15^C in Supplementary Fig. 2 after an overall scaling (~30%) compatible with the theoretical uncertainties on the absolute value. The calculations show an enhancement of the cross-section just above *S*_2n_, which is consistent with the experimental data, where a large structure at 10.5 MeV is present. The main contribution in this energy region was found to come from the absorption of the two neutrons. This means that a ^13^C+*n*+*n* resonant configuration can account for the observed structure at 10.5 MeV. However, the unknown bump at *E*_x_=13.7±0.1 MeV is not reproduced within this approach. Similar results were found for the ^12^C(^18^O,^16^O) reaction, where the bump at *E*_x_=16.9±0.1 MeV in the ^14^C spectrum was not described. This indicates that a more complete treatment of the target+*n*+*n* system with the inclusion of the *n*-*n* correlations would be required to describe such structures.

To test the effect of *n–n* correlations in the data analysis, the extreme hypotheses of the removal of a di-neutron (system with mass *A*=2, spin *S*=0 and isospin *T*=1) from ^18^O and freeze-out of the target degrees of freedom were adopted within the time-dependent Schroedinger equation approach^36^,^37^. For the case of ^15^C, the time-dependent Schroedinger equation was solved in a three-dimensional space using Woods–Saxon potentials of radii *r*=1.25 *A*^1/3^ and diffuseness *a*_0_=0.7 fm. It describes the evolution of a wavefunction initially in the target potential at rest when the projectile passes by at a given impact parameter. The resulting calculations predict the presence of a broad structure centred at ~12 MeV in the inclusive energy spectra, as shown in Supplementary Fig. 6. The absolute value of the calculated cross-section was scaled to ascertain the maximum contribution in the energy spectra coming from such a process. It is associated to the towing mode of the di-neutron removed from the ^18^O projectile. Our results show that neither such a mechanism is at the origin of the ^15^C bump at *E*_x_=13.7±0.1 MeV.

## Author contributions

F.C., D.C., M.C., A.C. and A.F. proposed the experiment. F.C., D.C., M.C., A.C. and A.F. developed and set-up the MAGNEX spectrometer for the experiments. F.C., D.C., M.C., M.B., C.A., F.A., A.C., A.F., S.F., R.L. and J.A.S. participated to the data taking. F.C., D.C and M.B. performed the data reduction. A.B., D.C. and M.B. performed break-up calculations with independent particle model. M.C. and J.A.S. performed towing mode calculations. E.K. and D.C. performed the cQRPA calculations. J.L. and F.C. calculated the absolute cross-sections. L.F., A.V., D.C. and M.B. extracted the strengths from the measured cross-sections. F.C., D.C. and M.C. wrote the manuscript. All the authors have revised the manuscript.

## Additional information

**How to cite this article**: Cappuzzello, F. *et al*. Signatures of the Giant Pairing Vibration in the ^14^C and ^15^C atomic nuclei. *Nat. Commun.* 6:6743 doi: 10.1038/ncomms7743 (2015).

## Supplementary Material

Supplementary Information Supplementary Figures 1-7, Supplementary Methods and Supplementary References

## Figures and Tables

**Table 1:** Main spectroscopic features of the populated states.

**S.No.**	**Excitation energy (MeV) (present work)**	**Excitation energy (MeV) (values from ref. 38)**	***J***^*π*^ (*)
*^15^C states*			
1	0.00±0.02	0	1/2^+^
2	0.73±0.02	0.7400	5/2^+^
3	3.12±0.02	3.103	1/2^−^
4	4.21±0.02	4.220	5/2^−^
5	4.65±0.02	4.657	3/2^−^
6	5.87±0.02	5.866	1/2^−^
7	6.85±0.02	6.841	7/2^−^
8	7.36±0.02	7.352	9/2^−^
9	8.47±0.02	8.47	1/2^+^, 3/2^+^, 5/2^+^ (from ref. 39)
10	9.06±0.02	9.00	
11	13.7±0.1		1/2^−^ (present work)
*^14^C states*			
1	0.00±0.02	0	0^+^
2	6.10±0.02	6.0938	1^−^
3	6.71±0.02	6.7282	3^−^
4	7.00±0.02	7.0120	2^+^
5	7.36±0.02	7.3414	2^−^
6	8.33±0.02	8.3179	2^+^
7	9.81±0.02	9.7460	0^+^
8	10.43±0.02	10.425, 10.498	2^+^, 3^−^
9	10.73±0.02	10.736	4^+^
10	12.88±0.02	12.963	3^−^
11	13.96±0.02	14.05	
12	16.42±0.02	16.43	6^+^ (from ref. 40)
13	16.74±0.02	16.715	6^−^ (from ref. 40)
14	16.9±0.1		0^+^ (present work)

^*^Values from ref. 38, except those explicitly indicated.
