# Supplementary material for: Signatures of the Giant Pairing Vibration in the 14C and 15C atomic nuclei
Source: Nat Commun. 2015 Mar 27;6:6743. doi: 10.1038/ncomms7743 (PMC4389259; doi:10.1038/ncomms7743)
Supplement: Supplementary Information — Supplementary Figures 1-7, Supplementary Methods and Supplementary References [file ncomms7743-s1.pdf]

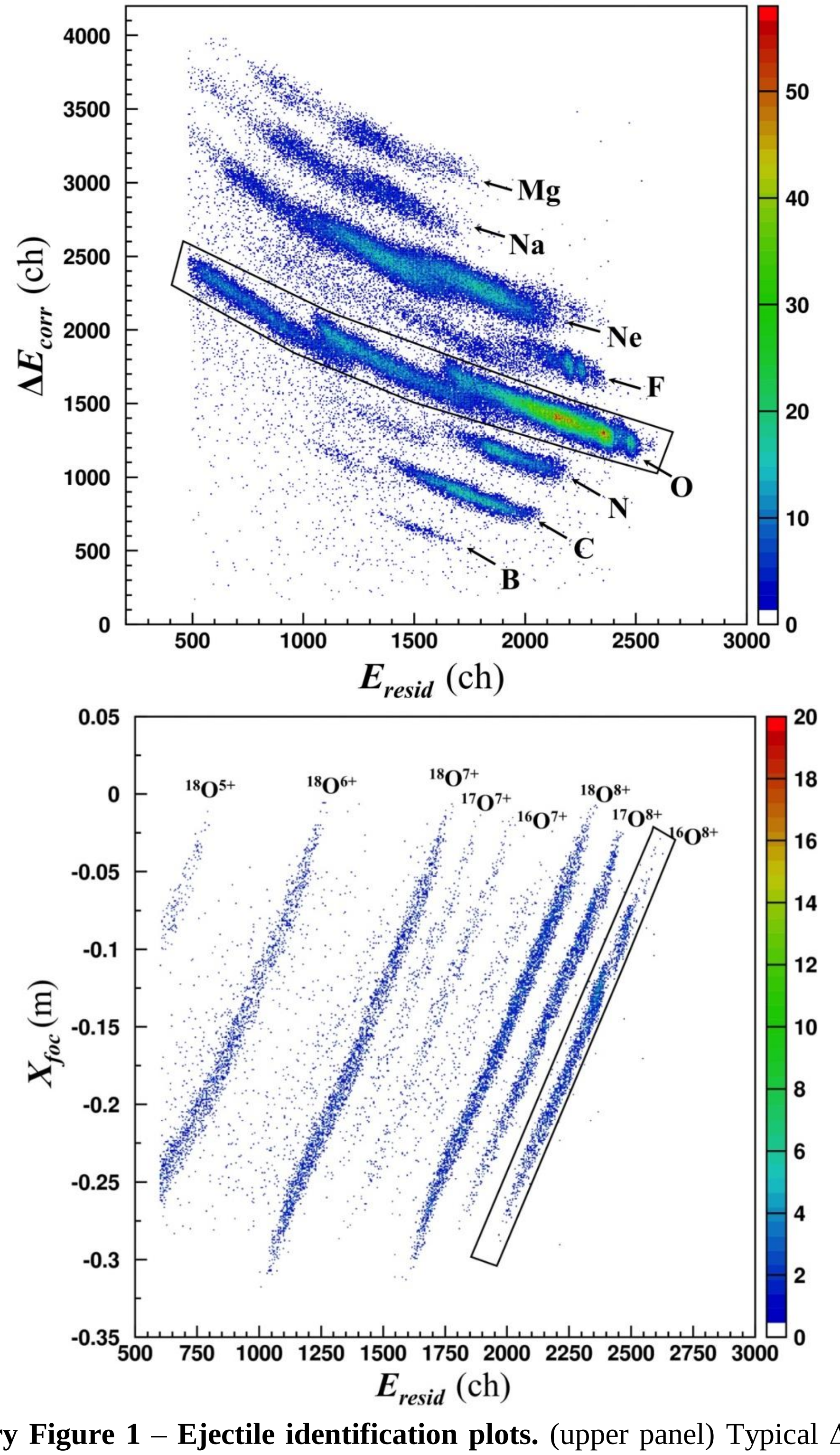

Supplementary Figure 1 – Ejectile identification plots. (upper panel) Typical ΔE_{corr} vs E_{resid} plot for the ejectiles detected in the reaction ^{18}O + ^{13}C at 84 MeV incident energy for a single silicon detector. The different ion species and the coarse graphical contour on the ^{16}O region are also indicated. (lower panel) Typical X_{foc}- E_{resid} matrix plotted with the graphical condition on the ΔE_{corr} - E_{resid}. The different Oxygen isotopes and the graphical contour selecting the $^{16}O^{8+}$ejectiles are also indicated.

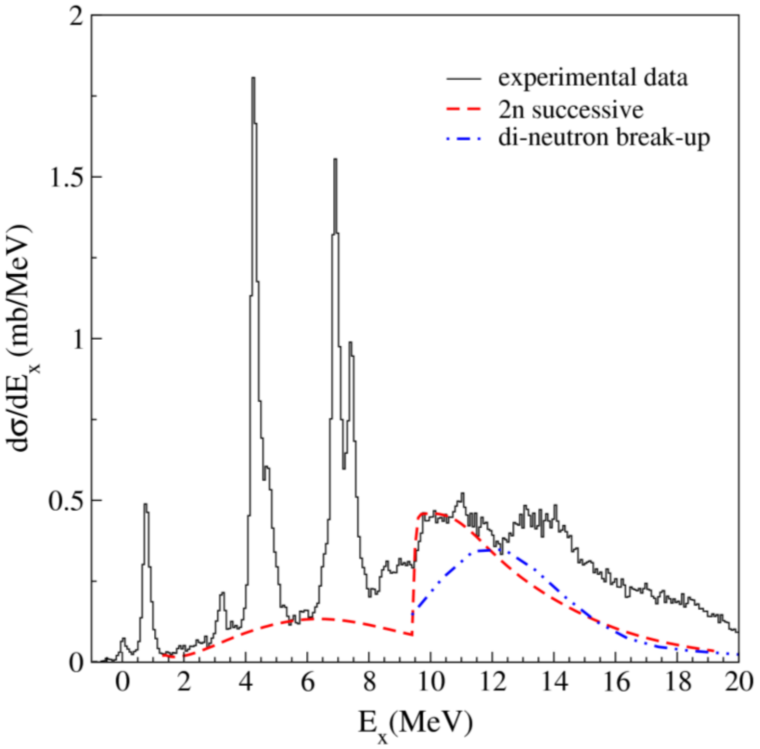

Supplementary Figure 2 - Projectile break-up calculations. Experimental inclusive energy spectrum for the $^{13}C(^{18}O,^{16}O)^{15}C$ reaction at 84 MeV and 7° < θ_{lab} < 17° (black line); independent removal of the two neutrons (red dashed line); di-neutron TDSE calculation (blue dot-dashed line).

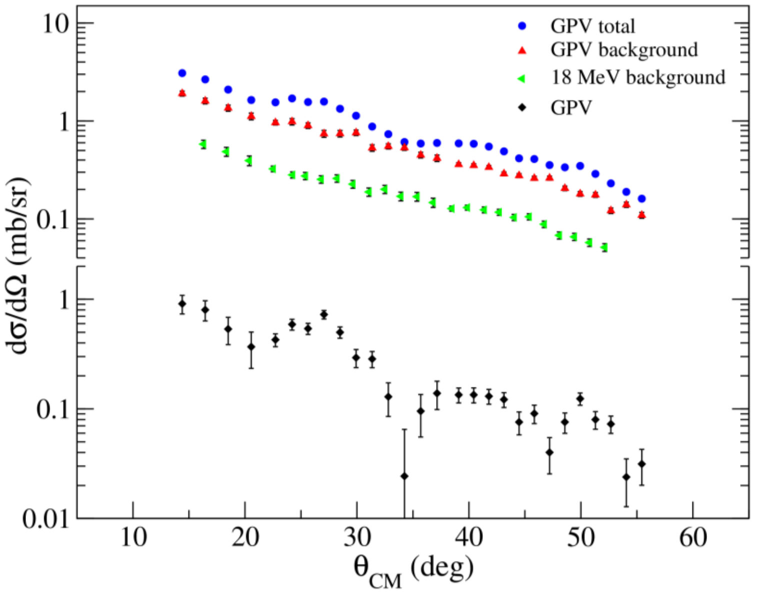

Supplementary Figure 3 – Background subtraction for the ^{15}C GPV angular distribution. Comparison between the GPV distribution without any background subtraction (blue circles), the subtracted background underneath the GPV (red up triangles), the background present in the region at 18 MeV (green left triangles) and the final GPV (black diamonds) angular distributions for the ^{15}C residual nucleus.

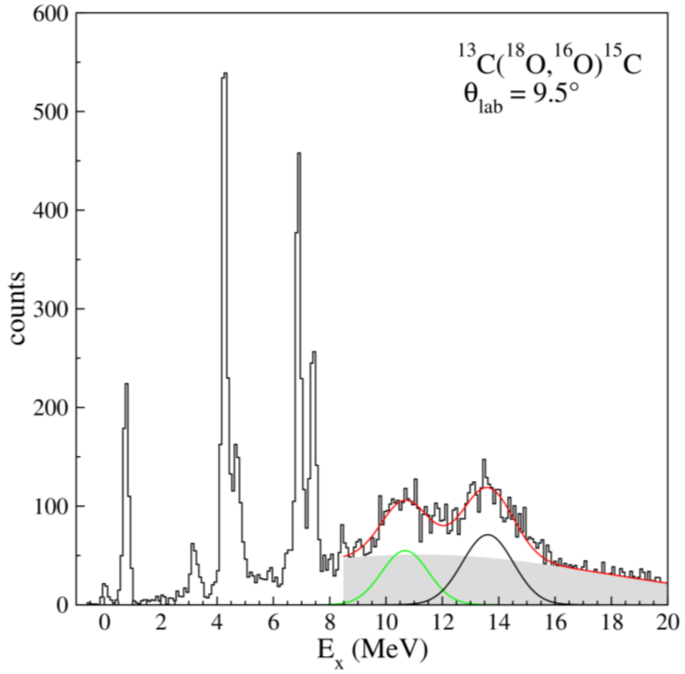

Supplementary Figure 4 – Second model for the background underneath the GPV. Energy spectrum for the $^{13}C(^{18}O,^{16}O)$ reaction. The second model assumed for the GPV background subtraction is shown with the grey area and the green Gaussian. The GPV is indicated as the black Gaussian.

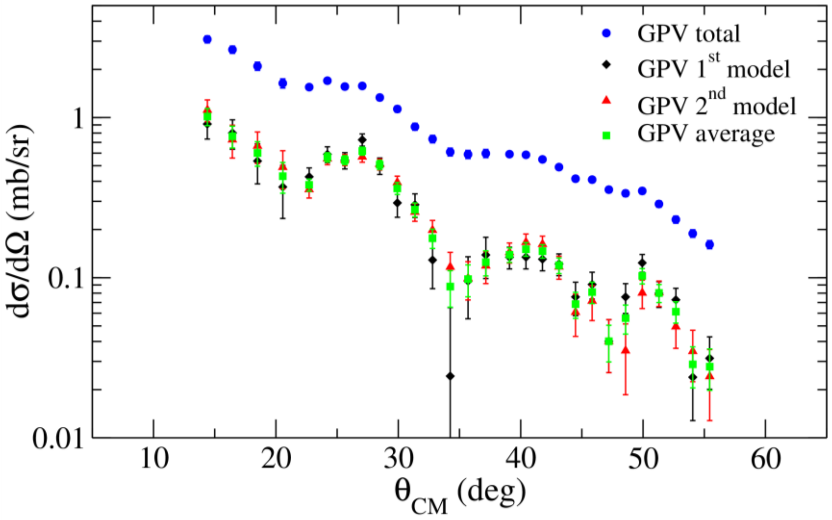

Supplementary Figure 5 – ^{15}C GPV angular distribution. Comparison between the ^{15}C GPV angular distribution obtained assuming two different models (black diamonds and red triangles) for the background subtraction and assuming no background (blues circles). The first model is shown in Figure 1 (right panel) of the main text and the second one is shown in Supplementary Figure 3. The weighted average between the two models is shown as the green squares.

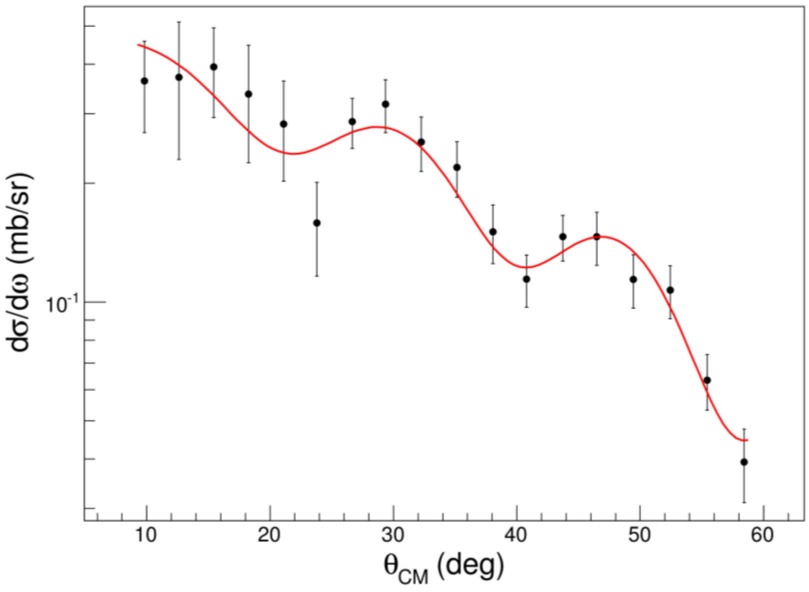

Supplementary Figure 6 – Oscillating pattern of the GPV angular distribution. Fit of the experimental ^{14}C GPV angular distribution by the model function *F* (see text).

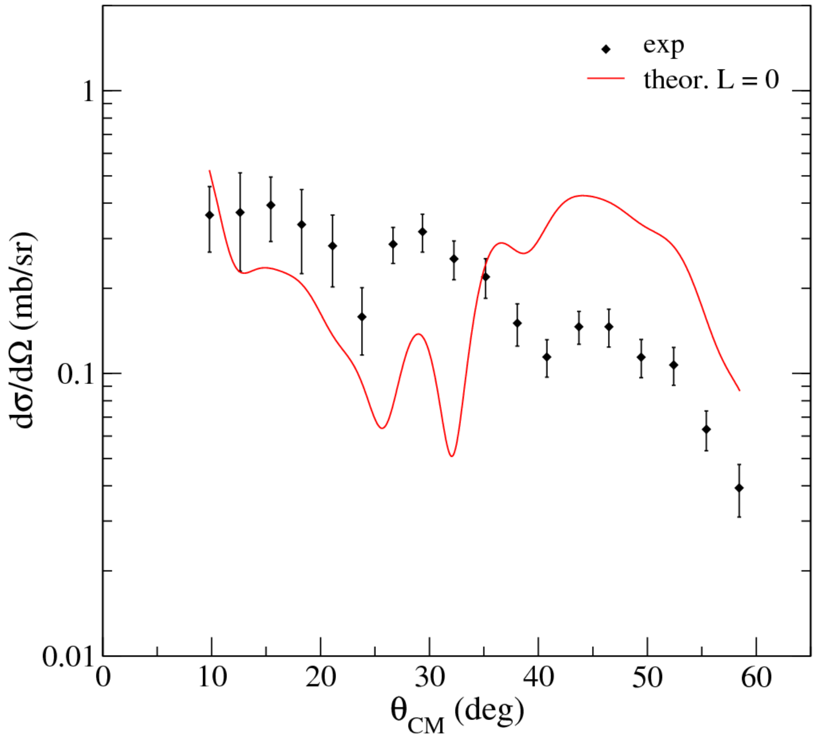

Supplementary Figure 7 – Comparison with calculations. Discretized continuum scheme calculations for the $L = 0$ case (red line) and experimental cross section angular distribution for the ^{14}C resonance at 16.9 ± 0.1 MeV. No scaling factors are used.

Supplementary Methods

Models for the background underneath the GPVs. The angular distributions of the absolute cross-section were deduced for the most intense transitions, including those populating the wide resonances observed above the two-neutron emission threshold. The differential solid angle for the full spectrometer acceptance was carefully determined taking into account the overall transport efficiency, as described in ref.[1]. A dead-time coefficient of ~30% was measured. An angular bin of 1° in the laboratory reference frame was chosen for the GPV angular distributions in order to achieve a good compromise between the statistical uncertainties in the number of counts, the background subtraction and the angular resolution.

The contribution to the angular distributions due to the continuous background in the spectra was estimated at each angle by a least-squared approach with a Gaussian model shape superimposed on a linear background as shown in Figure 1 of the main text. The adopted background model is consistent with two-neutron break-up calculations, performed considering an independent removal of the two neutrons, as described the Methods section. In order to carefully look at the projectile break-up contribution to the angular distribution, a comparison between the GPV angular distribution obtained without any background subtraction, the subtracted background and the final GPV distributions is shown in Supplementary Figure 3 in the case of ^{15}C. The oscillating pattern of the GPV angular distribution is slightly visible in the total one, since it is smoothed by the flat background underneath. Such a background has the same shape of that at about 18 MeV excitation energy (green left triangles in Supplementary Figure 3), where there are no resonances.

We used different models for the background subtraction and the obtained results for the centroid and width of the resonances and also the shape of the angular distributions did not change within the quoted uncertainties. As an example, a second model for the background subtraction is show in Supplementary Figure 4 , which assumes a wide Gaussian (centroid = 11.2 MeV, FWHM = 16 MeV) for the background underneath the GPV (grey area in Supplementary Figure 4) and a Gaussian model for the structure at ~ 11 MeV (centroid = 10.7 MeV, FWHM = 2 MeV) (green curve in Supplementary Figure 4). A comparison between the GPV angular distributions obtained using different background models is shown in Supplementary Figure 5 . The first model (black diamonds) assumes the background shown in Figure 1 of the main text, the second model (red triangles) is shown in Supplementary Figure 4 and the third one (blue circles) is the GPV angular distribution obtained without any background subtraction. The average between the two models is also shown (green squares). This comparison demonstrates that both the shape and the absolute value of the GPV cross section angular distribution are stable within the error bars.

Oscillating pattern of the GPV angular distributions. In order to quantify the presence of oscillations in the ^{14}C GPV angular distribution, a statistical analysis was performed. The angular distribution shape was compared to a model function composed by a cylindrical Bessel (J_0) plus a Gaussian:

$$F = aJ_0(kr)^2 + he^{-\theta_{\mathrm{CM}}^2/2\sigma^2}$$

where $k = \sqrt{k_{\mathrm{i}}^2 + k_{\mathrm{f}}^2 - 2k_{\mathrm{i}}k_{\mathrm{f}}\cos(\theta_{\mathrm{CM}})}$ is the wave number corresponding to the momentum transfer ($p = \hbar k$), r is connected to the average impact parameter at which the transfer takes place and a, h and σ are free parameters. Considering the present ^{12}C(^{18}O,^{16}O)^{14}C reaction at 84 MeV

incident energy, we obtain k_i = 3.4 MeV/c and k_f = 2.5 MeV/c. A value r = 4 fm was used. The resulting fit is shown in Supplementary Figure 6 . The values obtained for the free parameters were:

$a = 1.00 \pm 0.24$
$h = 0.31 \pm 0.04$
$\sigma = 29 \pm 9$

The a parameter is different from zero beyond 4 standard deviations confidence level, thus the contribution of the oscillating Bessel function is necessary in the model for describing the ^{14}C GPV angular distribution. This analysis shows that the oscillating shape of the ^{14}C GPV angular distribution is confidently established.

Cross section calculations within the discretized continuum scheme. In order to describe the cross section angular distribution of the ^{14}C resonance at 16.9 ± 0.1 MeV the discretized continuum scheme of ref.[2] was used. The extreme cluster model approximation for the two-neutron pair was adopted. Within such approximation, the two neutrons are paired anti-parallel and coupled to a zero intrinsic angular momentum (S = 0).

For the transfer to open states the cluster model corresponds to a 3-body calculation (projectile, target, neutron pair) which is an approximation of the actual 4-body problem (projectile, target, neutron, neutron). In the case of ^{6}He interaction with light and heavy targets it has been shown that the three-body model is a reasonable approximation[3,4] . However, the equivalence between the three-body and four-body methods has not been proven for systems with a larger two-neutron binding energy, as the ^{18}O projectile (S_{2n} (^{18}O) = 12.188 MeV, S_{2n} (^{6}He) = 0.971 MeV). For this reason fine details of the calculation should not be accounted for when such 4-body to 3-body projection is applied as in the present case.

A reasonable description of the full 3-body problem is that provided by the approach of ref.[2], where the continuum bins are derived from the Jacobi coordinates of the relative motion between the valence particle (x) and the target (T). This is assumed to be a valid approach for the description of the GPV since it corresponds to a resonance of the two-neutron + target system and lives long enough compared to the crossing time of the projectile during the reaction.

Replacing in the scattering amplitude the exact wave function by the elastic wave function[5,6] and using the prior representation, it is obtained[7]:

$$T_{prior} = \left\langle \psi_f^{(-)} \middle| V_{xT} + U_c - U_{pT} \middle| \phi_0 \chi_0 \right\rangle \tag{1}$$

where $\phi_0\chi_0$ are the asymptotic entrance channel wave functions and the final state $\psi_f^{(-)}$is the exact three-body wave function with incoming boundary conditions, expanded in terms of the $x + T$ continuum and bound (if any) states as

$$\psi_f^{(-)}(\boldsymbol{r},\boldsymbol{R}) = \sum_{\beta=0}^{N} \phi_\beta(\boldsymbol{r})\,\chi_\beta(\boldsymbol{R}) \tag{2}$$

where $\phi_\beta(\mathbf{r})$ represents the set of bin wave functions constructed as wave packets from pure scattering states[7,8,9]. This expansion goes beyond the Distorted Wave Born Approximation (DWBA) method since couplings between final states are also considered in the wave functions (2). In

expression (1) V_{xT} is the particle-target binding potential, U_c is the optical potential that describes the elastic scattering of the core by the target and U_{pT} is the projectile-target optical potential (cluster folding potential)[2]. It is important to note that the presence of the V_{xT} potential allows the description of the $x + T$ resonant excitations.

The parameter free double folding São Paulo real potential was used for the present calculations[10]. The imaginary part of the potential which fits the elastic scattering, when there is no relevant couplings to the elastic channel, is represented by 0.6 times its real part. Recently, it has been shown that this prescription is the most appropriate when all the important bound state couplings are explicitly taken into account and the dissipative reaction channels (like breakup channel, and/or deep inelastic channels, or any excitation of the continuum spectrum) are globally accounted for[11]. This value was assumed for the present U_{pT} optical potential. For the V_{xT} part, a Woods-Saxon potential was used with a reduced radius of 1.28 fm and diffuseness of 0.6 fm, the same as ref.[12] . The depth was varied in order to fit 2*n* binding energy as well as the position of the 16.9 ± 0.1 MeV resonance. In the coupling scheme the three 0^+ bound states of the ^{14}C were included (E_x = 6.589, 9.746, 16.9 MeV). The convergence of the calculation was obtained considering a maximum angular momentum of 100ħ and R_{max} = 600 fm for the ^{16}O and ^{14}C relative motion and a maximum r_{max} = 100 fm for the 2*n* -^{12}C bins integration. The bin width was set to 2 MeV to account for the experimental width value.

The resulting calculation, performed for $L = 0$, is superimposed to the experimental results in Supplementary Figure 7. The absolute value is consistent with the experimental one, without any scaling factor. The model space was also enlarged in order to check if the coupling to other continuum states might affect the final result for the resonant state. It was found that the inclusion of other continuum states and higher angular momenta did not considerably affect the results.

Supplementary References

1 Cavallaro, M. *et al.* Transport efficiency in large acceptance spectrometers. *Nucl. Inst. and Meth. A* **637**, 77-87 (2011).

2 Moro, A. M. & Nunes, F. M. Transfer to the continuum and breakup reactions. *Nucl. Phys. A* **767**, 138 (2006).

3 de Faria, P. N. *et al.* α-particle production in ^{6}He+^{120}Sn collisions. *Phys. Rev. C* **82**, 034602 (2010).

4 Pires, K. C. C. *et al.* Experimental study of ^{6}He+^{9}Be elastic scattering at low energies. *Phys. Rev. C* **83**, 064603 (2011).

5 Chatterjee, R., Banerjee, P. & Shyam, B. Projectile structure effects in the Coulomb breakup of one-neutron halo nuclei. *Nucl. Phys. A* **675**, 477 (2000).

6 Chatterjee, R., Shyam, R. Coulomb-nuclear interference in the breakup of ^{11}Be. *Phys. Rev. C* **66**, 061601 (2002).

7 Yahiro, M. *et al.* Effects of deuteron virtual breakup on deuteron elastic and inelastic scattering. *Prog. Theor. Phys. Suppl.* **89**, 32 (1986).

8 Austern, N. *et al.* Continuum-Discretized Coupled-Channels calculations for three-body models of deuteron-nucleus reactions. *Phys. Rep.* **154**, 125 (1987).

9 Yahiro, M., Nakano, M., Iseri, Y. & Kamimura, M. Coupled-Discretized-Continuum -Channels method for deuteron breakup reactions based on three-body model. *Prog. Theor. Phys.* **67**, 1464 (1982).

10 Chamon, L. C., Pereira, D., Hussein, M. S., Cândido Ribeiro, M. A. & Galetti, D. Nonlocal description of the nucleus-nucleus interaction. *Phys. Rev. Lett.* **79** , 5218-5221 (1997).

11 Pereira, D., Lubian, J., Oliveira, J. R. B., de Sousa, D. P. & Chamon, L. C. An imaginary potential with universal normalization for dissipative processes in heavy-ion reactions. *Phys. Lett. B* **679**, 330-335 (2009).

12 Cavallaro, M *et al.* Quantitative analysis of two-neutron correlations in the ^{12}C(^{18}O,^{16}O)^{14}C reaction. *Phys. Rev. C* **88**,054601 (2013).
